# Harnessing Clinical Trial Capacity to Mitigate Zoonotic Diseases: The Role of Expert Scientists in Ethiopia

**DOI:** 10.3389/fpubh.2021.621433

**Published:** 2021-04-01

**Authors:** Senait Belay, Mirutse Giday, Tsegahun Manyazewal

**Affiliations:** ^1^Center for Innovative Drug Development and Therapeutic Trials for Africa (CDT-Africa), College of Health Sciences, Addis Ababa University, Addis Ababa, Ethiopia; ^2^Faculty of Veterinary Medicine, Hawassa University, Hawassa, Ethiopia; ^3^Aklilu Lemma Institute of Pathobiology, Addis Ababa University, Addis Ababa, Ethiopia

**Keywords:** clinical trial, zoonotic disease, Ethiopia, one health, human resources

## Abstract

**Background:** The emergence and resurgence of zoonotic diseases have continued to be a major threat to global health and the economy. Developing countries are particularly vulnerable due to agricultural expansions and domestication of animals with humans. Scientifically sound clinical trials are important to find better ways to prevent, diagnose, and treat zoonotic diseases, while there is a lack of evidence to inform the clinical trials' capacity and practice in countries highly affected with the diseases. This study aimed to investigate expert scientists' perceptions and experiences in conducting clinical trials toward zoonotic diseases in Ethiopia.

**Methods:** This study employed a descriptive, qualitative study design. It included major academic and research institutions in Ethiopia that had active engagements in veterinary and public health researches. It included the National Veterinary Institute, the National Animal Health Diagnostic and Investigation Center, the College of Veterinary Medicine at Addis Ababa University, the Ethiopian Public Health Institute, the Armauer Hansen Research Institute, and the College of Health Sciences at Addis Ababa University. In-depth interviews were conducted with expert scientists. Data were collected from October 2019 to April 2020. Data analysis was undertaken using open code 4.03 for qualitative data analysis.

**Results:** Five major themes, with 18 sub-themes, emerged from the in-depth interviews. These were: challenges in the prevention, control, and treatment of zoonotic diseases; One Health approach to mitigate zoonotic diseases; personal and institutional experiences in conducting clinical trials on zoonotic diseases; barriers in conducting clinical trials toward zoonotic diseases; and strategies that promote conducting clinical trials on zoonotic diseases. Conducting clinical trials on zoonotic diseases in Ethiopia is hampered by a lack of clearly articulated ethics and regulatory frameworks, trial experts, financial resources, and good governance.

**Conclusion:** In Ethiopia, conducting clinical trials on zoonotic diseases deserves due attention. Strengthening institutional and human resources capacity is a pre-condition to harness effective implementation of clinical trials on zoonotic diseases in the country. In Ethiopia where skilled human resource is scarce, One Health approach has the potential to form multidisciplinary teams to systematically improve clinical trials capacity and outcomes in the country.

## Background

The importance of zoonotic diseases has increased at global and regional levels in recent years in connection with intensive human and wildlife migration, urbanization, human population growth, increased international travel and trade of animals and products of animal origin, and intensification of animal production (1–3). The emergence and resurgence of zoonotic diseases have continued to be a major threat to global health and the economy. Developing countries have a higher incidence and prevalence of zoonoses, and this is attributed to the lack of adequate control mechanisms, inadequate infrastructure, and inadequate information on their significance and distribution (4, 5). Many of the human diseases that are new, emerging, and re-emerging in these settings are caused by pathogens that originate in animals or products of animal.

Ethiopia has the second largest human population and the largest livestock population and is particularly vulnerable to the effects of zoonotic diseases because the economy is largely dependent on agriculture (6–8). Roughly 80% of households have direct contact with domestic animals, that favor an opportunity for infection and spread of disease (9). Ethiopia also ranks very high in the health burden of zoonotic diseases and in having a large population of poor livestock keepers (10). Since a large number of zoonotic diseases endemically arise in Ethiopia, a prioritization process was essential to categorize the most serious zoonotic diseases that should be co-operatively addressed by animal and public health agencies to exploit the impact on the health of people and animals. The zoonotic diseases prioritization workshop that was conducted in Ethiopia from September 25 to 26, 2019 guided a range of government agencies through the process to prioritize zoonotic diseases of greatest concern for Ethiopia and develop next steps and action plans. In the prioritization process, potential zoonotic diseases of concern were identified through a set of criteria. As a result of the prioritization process, human and animal health agencies co-operatively identified five zoonotic diseases they can jointly start to tackle. These are rabies, anthrax, brucellosis, Rift Valley fever, and zoonotic avian influenza (11).

Control of zoonotic diseases constitutes an important health matter. However, many factors are involved in prevention and control and it cannot be addressed by public health sectors only. Success in reducing the public health significance greatly depends on the level of cooperation between public and veterinary health sectors using one health approach in all aspects of the diagnosis, exchange of information, organization of shared surveillance systems, common training of staff and creation of community awareness. High-level commitment and the ability of national programmers to mobilize the necessary resources and to collaborate closely with other relevant sectors are needed to cope with the common challenges.

Conducting clinical trials toward zoonotic diseases is paramount to reduce the impact of zoonotic diseases on human and human health (12). It finds better ways to prevent, screen, diagnose, and treat the zoonotic diseases. In Ethiopia, the number of clinical trials that have been done keeps growing throughout the years. However, compared to other countries, it is still much lower. Africa, in general, shares only 2.4% of clinical trials generated globally and Ethiopia shares only 2.3% of clinical trials generated out of Africa and the trials are limited in number and variety (13–15). This is due to the existence of many challenges for the conduct of clinical trials that would determine the safety and efficacy of new products and interventions in the country. The tendencies to conduct clinical trials toward zoonotic disease in Ethiopia has not been studies. Thus, this study intended to investigate researchers' and experts' perceptions and experiences of conducting clinical trials toward zoonotic diseases in Ethiopia.

## Methods

### Study Setting and Design

This study employed a descriptive, qualitative study design. It included major academic and research institutions in Ethiopia that had active engagements in veterinary and public health researches. It included the National Veterinary Institute (NVI), the National Animal Health Diagnostic and Investigation Center (NAHDIC), the College of Veterinary Medicine at Addis Ababa University (CVM AAU), the Ethiopian Public Health Institute (EPHI), the Armauer Hansen Research Institute (AHRI), and the College of Health Sciences at Addis Ababa University (CHS AAU). The six institutions are all government-owned and have a long history providing research, academic, and disease prevention and treatment services in Ethiopia. NVI was established in 1964 and is currently certified by ISO/QMS to develop, manufacture, sell, and distribute veterinary vaccines as its primary mandate, and it is certified by ISO/IEC to function as a research and development laboratory for some specific laboratory tests (16). NAHDIC, established in 1995, is a national and East African reference laboratory for animal diseases and related public health problems. It has an ISO 17025 certified laboratory for molecular testing, including real-time PCR (17). EPHI works to improve the health of the general public of Ethiopia through undertaking research on priority health and nutrition issues for evidence-based information and technology transfer, effective public health emergency management, quality laboratory system, and training public health researchers and practitioners for pre-eminent public health interventions (18). CVM AAU, established in 1963, provides teaching, problem-solving research, and community services including treating all species of animals (over 20,000–25,000 patients every year), vaccination, consultancy, ambulatory clinic, artificial insemination, veterinary drug sale, harness making, and animal welfare education of school children among others (19). AHRI, founded in 1970, works to improve medical care, health, and well-being of the public by generating and delivering scientific evidence, developing new tools and methods through biomedical, clinical, and translational research, and to serve as a hub for technology transfer and capacity building in medical research and training (20). CHS AAU provides high quality and regionally relevant health sciences training, research and community services at both undergraduate and post-graduate levels. It is the only institution where some specialized tertiary healthcare services are given (21).

### Participants

We followed a purposive sampling method to recruit the participants from the selected study areas. The total number of participants in the study was 14 using data saturation procedures. The Inclusion criteria were holding a proven exhibit primarily leading research activities or research units within the institutions, having a minimum of three publications as the first author, and/or a minimum of 5-year work experience in the research or management of zoonotic research. According to the legal procedure in the institutions, institutional directors, or college deans at each institution should first give verbal approval for a researcher to proceed to contact a participant. Therefore, after getting an approval letter for the study from the Addis Ababa University College of Health Science, CDT-Africa, we contacted institutional directors or college deans at each institution to select the participants. All of the institutional directors or college deans received the letters and gave their verbal approval. Some recruited the participants themselves based on the information they read in the letter, whereas, others allowed us to select by ourselves. Finally, the selected participants agreed and participated in the study.

### Data Collection

We conducted a face-to-face, open-ended in-depth interviews to allow participants to discuss their opinions, views, and experiences fully in detail. We prepared a list of questions to be responded to by each participant. All the interviews were m by the primary investigator during work hours in private rooms and the discussions were open for any questions or clarification. The participants themselves scheduled when and where they were to be interviewed, and reported that they were comfortable about their decisions. The language of communication with the participants during the in-depth interviews was English. We conducted an in-depth interview with two researchers to test the validity of the interview questions and gain the necessary experience. When the primary investigator conducted in-depth interviews, she audio-recorded the interviews for later transcription, and she took additional notes. The use of audio records determined by the participants' consent and each of them was asked to and agreed to be recorded, except two who allowed taking notes only. The interviews had nine open-ended questions, uniquely developed for the sole purpose of this study.

### Data Analysis and Interpretation

We started analyzing the material during the fieldwork to identify gaps early and produce a good description of topics. The audio files from the in-depth interviews were transcribed and data analysis was undertaken using open code 4.03 for qualitative data analysis (22). To improve the rigor of the study, two investigators were involved in the data analysis. Braun and Clark's reflexive thematic analysis framework (23) was used and the analysis process followed the six phases: familiarization, initial coding, theme construction, reviewing themes, defining themes, and producing the report.

### Ethical Clearance and Consideration

Ethical clearance was obtained from the Scientific and Ethics Review Committee of the Centre for Innovative Drug Development and Therapeutic Trials for Africa (CDT-Africa), College of Health Sciences, Addis Ababa University. Letters of ethical clearance as well as a letter of support were submitted to the selected academic and research institutions and researchers, and their consent was obtained. Interviewees were briefed on the purpose of the study without any form of deception before securing informed consent from them and confidentiality maintained entirely.

## Results

### Demography of the Participants

In total, 14 participants were involved, including two from NVI, two from CVM AAU, two from NAHDIC, four from AHARI, three from CHS AAU, and one from EPHI. Table 1 summarizes the participants' demographic characteristics.

**Table 1:** Participants demographic information.

**Participant ID**	**Name of the institution**	**Highest level of education**	**Academic rank/Position in the institution**	**Service year**	**Publication as the first Author**
CDP001	AHRI	PhD	Research directorate	15 year	18
CDP002	CVM AAU	PhD	Professor	25 year	50
CDP003	EPHI	MSc	Research directorate	10 year	16
CDP004	NVI	PhD	Drug formulation director directorate	18 years	8
CDP005	NAHDIC	MSc	Senior researcher	13 years	6
CDP006	CHS AAU	PhD	Professor	30 years	60
CDP007	AHRI	MSc	Physician	8 years	4
CDP008	AHRI	MSc	Principal Investigator	18 years	15
CDP009	AHRI	PhD	Head of the one health	15 years	20
CDP010	NVI	PhD	Senior researcher	13 years	7
CDP011	CVM AAU	PhD	Associate Professor	18 years	20
CDP012	CHS AAU	PhD	Associate Professor /east Africa one health initiative director	21 year	56
CDP013	CHS AAU	PhD	Associate Professor	10 year	39
CDP014	NAHDIC	MSc	Senior researcher	9 years	4

### Study Themes

During the interview sessions, participants reflected upon several concepts around zoonotic diseases, clinical trials, one health approach, and integrative practices of the public and animal health system. Participants were also able to ground these discussions by drawing connections to their experiences in clinical research on zoonotic diseases and how these ideas may have influenced the research world. Many participants in this study reported their appreciation for the opportunity to participate in the interview as the topic is new with an appreciable idea. Some mentioned that the researchers allowed them to talk about issues that are always in their minds and planned to do more in the future. As such, five themes were identified from the data, each containing several sub-themes (Table 2). These themes are:-

1. Challenges in the prevention, control, and treatment of zoonotic diseases
2. One health approach to mitigate zoonotic diseases
3. Personal and institutional experiences in conducting clinical trials on zoonotic diseases.
4. Barriers in conducting clinical trials toward zoonotic diseases.
5. Strategies that promote conducting clinical trials toward zoonotic diseases.

**Table 2:** List of the themes and sub-themes (codes) that resulted from the data analysis.

**Themes**	**Sub-themes/Categories/codes**
Theme 1: Challenges in the prevention, control, and treatment of zoonotic diseases	1.1 lack of integration between public and veterinary health professionals and sectors, 1.2 Human capacity (lack of skilled personnel, lack of awareness and motivation) 1.3 Inadequate financial and other resources support (lack of funding, inadequate laboratory equipment, lack of infrastructure) 1.4 Limited research, experience (Lack of research materials/facilities, lack of conducive research atmosphere) 1.5 Problems related to awareness and attitude.
Theme 2: One Health approach to mitigate zoonotic diseases	2.1 Implementation of one health 2.2 Silo effect 2.3 Importance of clinical trial and other basic or applied researches intended for zoonotic diseases
Theme 3: Individual and institutional experiences in conducting clinical trials on zoonotic diseases	3.1 Personal experiences 3.2 Institutional experiences
Theme 4: Barriers in conducting clinical trials toward zoonotic diseases	4.1 Ethical and regulatory systems (delay of approval decision, complex and strict ethical and regulatory system) 4.2 Human resources (lack of skilled personnel, lack of awareness and motivation) 4.3 Administrative support, (unsupportive administrative system) 4.4 Budget and infrastructure (lack of funding, inadequate laboratory equipment, lack of infrastructure) 4.5 lack of time
Theme 5: Strategies that promote conducting clinical trials on zoonotic diseases	5.1 Capacity building 5.2 Developing clinical trial industries 5.3 Promoting other applied researches

#### Theme 1: Challenges in the Prevention, Control, and Treatment of Zoonotic Diseases

The challenges in the prevention, control, and treatment of zoonotic diseases are mentioned in many of the interviews. This theme and its five subthemes and some codes captured participants' perceptions and experiences on the challenges in the prevention, control, and treatment of zoonotic diseases. The codes (subthemes) supported by descriptions and quotes are as follows:

##### Lack of Integration Between Human and Animal Health Professionals and Sectors

All participants perceived a lack of integration between human and animal health professionals as the main challenge. They mentioned that people who were engaged in the public health profession did not emphasize zoonotic diseases; they did not have the interest to get involved in research concerning zoonotic diseases. They considered dealing with the zoonotic related issue was the responsibility of animal health professionals. Moreover, participants stated that researchers who were professionals in veterinary health and those experts in public health were not collaborating to do research together. Even geographically public health institutes and veterinary health institutes were not in the same area.

**“***Researchers who are working in the field of veterinary health and those who are working in human health are not working collaboratively. That is the most difficult challenge…for example, if you take Addis Ababa University, College of Veterinary Medicine, it is in Bishoftu but the College of Health Sciences in Tikur Anbesa….even geography can be also a problem to work together”(CDP013)*. ”*…I mean … people who are engaged in public health, they don't have Knowledge of zoonotic diseases… You know they consider this responsibility is for veterinarians…. They don't give much attention to zoonotic diseases …. That is a major challenge” (CDP010)*.

##### Lack of Trained Manpower and Inadequate Finance

On the other hand, lack of human capacity, limited resources, and other related tools were reported by the majority of participants as challenges in the prevention, control, and treatment of zoonotic diseases. They mentioned that lack of skilled manpower who specialized in the area of zoonotic diseases with knowledge of different aspects of those diseases required to propose, initiate, and conduct of any research intended for zoonotic diseases. Financial scarcity also another challenge in Ethiopia as it is also in most low-income countries; research is considered to be a luxury because of economic constraints. Participants indicated that as long as we do not have experts in the area who can deal with zoonotic diseases and well-organized financial systems, it could be difficult to prevent and control zoonotic diseases.

“*….I think yes… there are several challenges, in terms of human capacity who are specialized in the field and in terms of resource both financial and another resource…”(CDP012) “…Because we don't have tools to deal with the infection, to produce drugs such as vaccines, and so on…Even the diagnosis is very difficult for most of these diseases…they need a special laboratory for example diseases that needs complicated diagnostic techniques like brucellosis, anthrax. They need level 3 laboratories, but we do not have these …” (CDP007)*.

##### Limited Research

Others described that there is a lack of baseline data due to limited research in the area and could lead to the irrational use of drugs and the scarcity of vaccines to prevent those diseases. Several neglected zoonotic diseases are not yet researched in Ethiopia.

“*……The major challenges are related to issues of limited research in the area of zoonotic diseases and the knowledge and awareness of the community …” (CDP004)*.

##### Lack of Awareness and Attitude

Besides, lack of knowledge, attitude, and awareness in the society aggravate disease transmission between animals and humans as Ethiopia, a country that has the biggest herd of livestock in Africa where its economy largely depended on agriculture and most of the society lives closer to its animals. People don't know about those diseases and are not aware of how they get diseases from animals.

“*….We have the biggest herd of livestock in Africa and people are living very close to livestock especially in the rural areas; in the whole pastoral area, there is a close link between animals and people and increased human and animal contact leads to a risk of a high prevalence of zoonotic diseases in the area … I think this happens due to lack of awareness and scarcity of information in Ethiopia on this topic….”(CDP011)*.

#### Theme 2: One Health and Research Approach Intended for Zoonotic Diseases

This theme reflects the researchers' perceptions and experiences in connection with one health approach and different issues related to clinical trials and other basic or applied researches toward zoonotic diseases. It is divided into three further subthemes: implementation of one health, silo effect and importance of clinical trial and other applied researches intended for zoonotic diseases.

##### Implementation of One Health

All fourteen participants considered that the implementation of one health in Ethiopia would be a beneficial approach in reducing the burden of zoonotic diseases. They considered that one health would enable earlier detection of zoonotic diseases, and therefore earlier reporting and opportunities for prevention or management of zoonotic transmission from animals to humans. They were asked about their perceptions concerning one health approach and whether they had participated in any project, workshop, conference, and other duties associated with one health initiative. Data suggest that some of them were involved in one health initiative activities and observed challenges and opportunities to implement it. As it is a new concept in Ethiopia, the progress of implementation so far is somehow appreciable, as different ministries are preparing a platform that helps them work together. Other participants also discussed that although some institutions started participating in one health initiative; still there is no proper health implementation in Ethiopia. The participants' further stated that where we have several emerging and re-emerging pathogens, working in isolation on the veterinary pathogen or human pathogens will not lead us any further in the control of zoonotic diseases.

“*…To my knowledge, there is no proper health implementation in Ethiopia. We have a project in the Somali region. We are starting to implement one health initiative, but there is no result to show yet…. In my experience I am seeing that we still are not considering the whole ecosystem and the wildlife as part of the one health approach We started implementing one health but we have not yet exploited the full potential ….The research institute at which I am working, i.e. AHRI, is also involved in one health initiative and it has one health unit that focuses on addressing zoonotic diseases particularly related to bovine tuberculosis and other diseases of interest like Pasteurella, brucellosis and other bacterial diseases” (CDP003). “….in the case of one health approach,… it is a good idea to improve the diagnosis as well as the controlling and preventing method of transmission from animal to human and vice versa and there is a start of one health approach in Ethiopia in which we are just participating with other institutes working on this.…. Our institution (NAHDIC) as one member is participating in prioritizing zoonotic diseases using one health approach….” (CDP002)*.

##### Silo Effect

Participants discussed the issue that there were challenges in implementing one health approach. The majority of them mentioned the existence of the silo effect in the implementation of one health and have similar challenges to collaboratively prevent and control zoonotic diseases. Silo Effect in one health refers to lack of information flowing between institutions or parts of an organization and limits the interactions between members of different sectors, thus leading to reduced productivity and capacity of collaborative working.

“*….the Silo effect is the first and the most important challenge in the implementation of one health….you know there is no ministry or no office that works on one health …….Since it doesn*'*t belong to one ministry people have their circle and work on that circle and there is a very bad history of working together….as I said the challenges that are faced on issues around working on zoonotic disease are also faced encountered in one health program. ……” (CDP012)*.

##### Importance of Clinical Trial and Other Basic or Applied Researches Intended for Zoonotic Diseases

The significance of conducting clinical trials over other basic or applied researches was reported by most of the participants. In this regard, they mentioned that clinical trials had a broad concept. They stated that it was not only developing a new treatment but rather it included so many aspects it *is beyond that; a clinical trial is about vaccine, clinical trial is about diagnostics, a clinical trial is about lifestyle*. They further mentioned that as there was evidence of a gap in the control and prevention of zoonotic diseases in Ethiopia and thus a need for a new intervention in terms of developing new diagnostic tools, vaccines, or treatment.

“*….Yes,… because the evidence is always good.I mean my work was involving both clinical trial and leadership of one health partnership. First, we should start by defining and understanding what a clinical trial is. In this perspective, someone is considering clinical trial in the context of developing a new drug but a clinical trial is beyond that; a clinical trial is about. vaccine, clinical trial is about diagnostics, a clinical trial is about any intervention.if we think of for example a trial on vaccine efficacy and safety in this country for several diseases like rabies, do we work on and invest in zoonotic diseases vaccine development, diagnostic development…yes. Clearly… yes. As I said earlier, these are diseases that are highly affecting developing countries Like Ethiopia. The problem is ours and the solution should come from us. …For example, brucellosis is one of the vaccine-preventable diseases. Anthrax is one that is vaccine-preventable. Rabies is one of the vaccine-preventable diseases. So to answer your question, do we need a clinical trial for those diseases in Ethiopia.…” (CDP012). “….Yes, a clinical trial is more important than other basic or applied researches because you do have better, tangible information because a clinical trial is you know it is a method to evaluate the certain drug, or biological things, any kind of intervention have to first evaluate Before putting the ground.…. (CDP005)*.

In contrast, some participants reported that as the magnitude of most of the zoonotic diseases was not well-researched in Ethiopia, where there are inadequate epidemiological and other types of data, conducting clinical trial was not applicable. They thus further suggested that emphasis first be given to other basic or applied studies rather than to clinical trials.

“*….For some diseases that we just, don*'*t know enough, basic epidemiological studies would be more important to get a proper idea of what is going on. it may not be the right time to do clinical trials for these diseases because to me we are very far away from knowing the magnitude of these diseases like the rift valley or rabies. but I don*'*t think we know what the magnitude of those diseases is. The first thing is we need to do more work in integration beyond just simply talking about one health. We need to go the grass-root level and get more involved in basic researches…” (CDP009). “… Conducting clinical trials is good. I am not against that but I don*'*t give priority to a clinical trial on the zoonotic disease rather the basic researches. If you do basic researches on zoonotic diseases we can generate evidence. We can do awareness creation works on zoonotic diseases and.I prefer the basic and epidemiological researches on zoonotic diseases than clinical ….Probably it might be because I don*'*t know the magnitude of the problem but before doing a clinical trial, it is good to have adequate information on the burden of the diseases…For instance, understanding the basic characteristics of a pathogen at ultrastructure”(CDP008)*.

#### Theme 3: Personal and Individual Experiences in Conducting Clinical Trials on Zoonotic Diseases

Under this theme, participants discussed different issues related to their experiences in connection with clinical trials toward zoonotic diseases as well as institutional level perceptions and experiences.

Concerning personal and institutional experiences, participants claimed that there was a limited experience in conducting clinical trials in Ethiopia in general. The majority of the participants stated that they had never participated in any clinical trial toward zoonotic diseases due to some country-level challenges, such as institutional administrative problems and lack of funding opportunities.

“*…I have no experience in conducting clinical trials for zoonotic diseases. probably one reason is as I said we still do not have much knowledge on the epidemiology of those diseases. The second thing is a matter of prioritization of government and international bodies…As an institution, yes; I would say it is very low, to be frank. AHRI is more, I can say, working on clinical trial but when you come to specifically zoonotic diseases, it is very low. Like I said, usually, sponsors are interested in other diseases like HIV, TB, and Malaria than zoonotic diseases …” (CDP010)*.

On the other hand, few participants reported that they had the experience of conducting clinical trial toward zoonotic diseases involving tuberculosis, leishmaniasis which included vaccine trial, diagnostic trial, and an on-going treatment trial.

“*……ya so, this is the issue so, for example, I did with leishmaniasis And you know that leishmaniasis is a zoonotic disease and therefore we have been conducting a clinical trial. I was involved in at least 7 or 8 clinical trials. Some are completed and some on-going….so we have new formulation and resumption to the existing treatment are to evaluated we had to come up with the short duration of treatment this has been done and even the guideline. The treatment guideline has been changed as a result of that and there has been also a negative result. Because I said close to 8 clinical trials I was engaged at least one study which was interrupted because the result was discouraging … not always to be successful and but most of the time we were successful the same thing. It was a treatment trial and evaluation of diagnostic tests … a couple of diagnostic tests evaluated now the tests are in use in the health system. The result was published and has been translated and public health use in diagnostic and treatment now. in the movement, we have one clinical trial which is vaccines for leishmaniasis, but that is on-going…” (CDP006)*.

#### Theme 4: Barriers in Conducting Clinical Trials Toward Zoonotic Diseases

Participants outlined the different barriers they faced or perceived during their research life. Most of the barriers they mentioned were similar to the challenges faced in the prevention and control of zoonotic diseases except for a few of them. The identified ones were barriers related to the handling of ethical and regulatory systems, lack of human capacity, administrative problems, financial scarcity, and other resources for conducting clinical trials. Barriers may differ generally depending on the setting in which the clinical trials are conducted.

##### Barriers Related to Ethical and Regulatory Systems

The majority of participants indicated difficulties with ethical and regulatory requirements, either at a national or institutional level as the main barrier. Respondents claimed that approval of proposals takes a long time to get approval and that discouraged most researchers to think of and conducting clinical trials on zoonotic diseases.

“*… Ya…the main challenges for conducting a clinical trial is ethical clearance and regulatory approval especially in our country Regulators and the ethical clearance are most of the time delay and make the research lose interest…” (CDP005)*.

##### Financial Constraints and Other Resources

Most respondents report that clinical trial by itself is complicated. It is costly; it needs a long duration of time and organized documentation. They indicated that in the context of our country, as a developing country, most of the research institutions focus on basic or applied researches which is not financially expensive. Participants highlighted barriers related to infrastructure like lack of standard laboratory to conduct clinical trials was the most frequently cited resource limitation. They indicated that zoonotic diseases posed risk to people and the environment and thus a need for good laboratory facilities, which is not available in the country. The quote from one participant could be taken as evidence that a good laboratory facility is very crucial to conduct the different phases of a clinical trial for zoonotic diseases. Lack of skilled manpower was also the most frequently cited barrier to conduct clinical trials toward zoonotic diseases.

“*… it is costly, it takes time, it*'*s complicated …. and it needs a lot of good practice and good laboratory practice and documentation and ethical clearance also the problem …. you don't get timely feedback, or approvals from ethics committees and that takes time so the protocol would take up to 2 years to get it approval” (CDP006). “….the human and financial capacities are the main challenges, for example, 5 years ago I was engaging in a project with the main target of building capacity in all East Africa to conduct clinical trial so. Ethiopia was a member and the other country, Kenya, Tanzania, and Uganda. the idea was to build capacity then AHARI was identified as sister institutions that probably to upgrade to be a site where we conduct a clinical trial. So we bought very few basic equipments like freezers, incubators, so on but after a couple of years everything was stopped because of a lack of capacity to run the project… so at this time, we can*'*t even host phase two and other phases of clinical trial… probably we can do just phase one small clinical trial because of lack of capacity to do further phases of a clinical trial” (CDP008)*.

##### Barrier Related to Administrative Issue

Participants stated that a lack of support and encouragement from an institution is also another barrier. They reported failure to acquire grants as most of the grants for clinical trials were too competitive. Institutional grants were always keen to basic or applied researches and that resulted in a lack of motivation from research coordinators and administrative bodies. Certain quotations below show these and related opinions:

“*…support and encouragement from an institution is the main challenge because institutions give apriority for other epidemiological research rather than clinical trial … For example. I have submitted a protocol for the university thematic research grant in the genetics and behavior related topic…which was…the effect of genetics on behavior so, we want to do a clinical trial on twins, but finally it got rejected because it's a kind of a clinical trial, basic research people assume that it is a kind of luxury so, getting institutional support was a big challenge” (CDP0*13). *The other problem is administrative support especially from the institutions is limited ….in clinical trial organizational support is crucial… there is reporting for example within 24 hours you know taking immediate action it might have emergencies … It needs support from the administrative but that is not available and also There is the issue of import of goods and supplies, medication and so on” (CDP006)*.

#### Theme 5: Strategies that Promote Conducting Clinical Trials Toward Zoonotic Diseases

Different suggestions were made by respondents on how to improve the conduct of clinical trials toward zoonotic diseases. Most of the suggestions were related to the challenges that have been identified above. Below are some of the common responses:

##### Capacity Building

The most common promoting factors identified by the study participants included capacity building including change of education system since Ethiopian scientists are not that much aware of and want to do clinical trials. There must be some reform in the educational system to address the need for clinical trials. Higher learning institutions could incorporate modules on clinical trials within their teaching programs for health professionals. The other factor mentioned by participants was related to capacity building of researchers by creating a platform to bring all different researchers together to conduct a clinical trial, which according to them, needs a lot of support to move forward, including from that of funding organizations, and policy and decision-makers. Participants from CVM AAU and CHS AAU mentioned that the following:

“*…ya…. So what could be done and I think, to begin with, education Not many scientists in Ethiopia are aware and wanting to do clinical trials, there must be some platform whereby …you know to address the need of clinical trial not only zoonotic diseases but also other diseases. I know there is some initiative in CDT Africa….to promote there is an approach to doing it as educational system.the higher learning institutions could incorporate modules about clinical trial within their teaching program and as far as I know, there is none as the movement so, you know health professionals in general before they graduated they should get a module of a clinical trial in the final year then they will get something about clinical research …they will get something about ethics and other related issues of clinical trial ….so that is a missing link and the ministry of education and higher education if they can address it which means we will have everyone comes out of school will have an idea of what clinical trial and some might decide to do clinical trials and so have it as a career and I think with that you know the scenario might change for the future*, for example, *EPHI they have a unit for zoonotic disease and also … Aklilulema institute of pathobiology ….those institutions should be leading this exercise otherwise it becomes an issue for just an interested individual, but there must be an institutional commitment they have to incorporate clinical trials in their programs and this message has to be conveyed to those institute” (CDP006). “ya…one thing we have to improve the capacity of researchers and funding organization they have to make open their doors for such kind of and also policymakers, Decision makers they have to be open to using outputs of clinical trials that conducted. There should be a kind of change of perception about what clinical trial is. the other one, there should be a kind of platform to bring all different professionals to work together in conducting clinical trials for these diseases unless We work together. Clinical trial for zoonotic diseases may not be successful as need.” (CDP011)*.

##### Developing Clinical Trial Industries

Other respondents talked about promoting factors in terms of infrastructure and locally developed clinical trial industry to motivate researchers to do clinical trials. The respondents reported that external partners only supported the conduct of a trial that focused on diseases of their interest but not zoonotic diseases that were relevant to the country. The respondents further stressed on the importance of locally developed tools to be self-sufficient in everything.

“*. I think the first thing is they have to need to do more on capacity building in human and also the infrastructure that is initial thing otherwise I believe Ethiopia is a good site for clinical trial what we are lacking is the human capacity on the other hand. I don't see the particular challenge and particular Solution for zoonotic diseases. it*'*s the same as any other diseases so generally in the clinical trial industry because of we don*'*t have locally Developed tools eternal partners are influenced us ….so we have to be self-sufficient by everything, the local scientific Community development should be We should have new tools and idea we going to do clinical trial” (CDP007)*.

##### Promoting Other Applied Researches

On the other hand, some respondents indicated that to promote conducting clinical trials toward zoonotic diseases, there was a need to first promote epidemiological researches before thinking of conducting clinical trials because of the need to understand the magnitude and the importance of diseases in our country. So we need to have the basic information of the diseases regarding magnitude and epidemiology.

“*To promote clinical trial.i don*'*t think. It is you know we are on the time to implement clinical trial.it should be established that we have to have to the ground otherwise it should. The country should have some industry working on pharma industry as well as biological things b/c we have to have our capacity rather than importing as a product so that if we are plan to produce or develop drugs, vaccines even we have to change our traditional medicines to market to make the marketable we have to conduct a clinical trial, so it*'*s the time also force us to establish this think we are on the right direction, the government Addis Ababa university. Open this clinical trial-related discipline so it is nice one awarded people to participate in clinical trial”. (CDP004)*.

## Discussion

In this study, in-depth interviews with 14 participants provided information on researchers' perceptions and experiences in conducting clinical trials toward zoonotic diseases, and several issues around zoonotic diseases were mentioned. Participants reported so many challenges in the prevention and control of these diseases in Ethiopia. The main challenge for the effective implementation of zoonoses control reported was the lack of integration between human and animal health professionals. Sectors working on public health, veterinary health, agriculture, and the environment must be aware of the importance to get involved in cooperative efforts in the control of zoonotic diseases. This finding is supported by many studies around the globe, which state the challenges of preventing and control of zoonotic diseases greatly due to lack of cooperation between medical and veterinary sectors in the diagnosis of zoonoses, exchange of information, organization of shared surveillance systems, common training of staff and creation of community awareness (24–27).

An additional challenge mentioned by the respondents was a lack of infrastructure and other resources, such as good laboratory facilities. Participants stated that zoonotic diseases needed at most care while researching because they pose a high risky for humans as well as the environment. The respondents stressed that there was a lack of good laboratory facilities, such as level three and above. Participants believed that funding could become a sensitive issue to conduct clinical trials on zoonotic diseases as such, mostly a problem of developing countries, like Ethiopia. Funding for clinical trials in a developing country comes mostly from Western countries in which pharmaceutical companies are based. In most low-income countries, research is a luxury because of economic constraints. This result was in line with studies reported elsewhere in different African countries (27, 28).

Participant emphasized the significance of one health approach, an interdisciplinary approach to zoonotic diseases, which encourages structured collaboration and coordination between human and animal health sectors, which have also been supported and highlighted in many other studies (29–31). The existence of the “silo effect” between professional sectors was also evident from the findings in this study. Participants perceived that the “silo effect” can inhibit cross-sectional collaboration and communication. This phenomenon has also been reported by several other studies (32, 33). The COVID-19 outbreak is evidence of how emerging animal-source of diseases cause a human health hazard globally (34, 35) and disrupt the healthcare system (36).

Participants stated that highly qualified personnel are required to propose, initiate, and conduct clinical trials. Such human resource development requires relatively stable, well-resourced research and higher education institutes, and well-established science governance systems, which is not the case in Ethiopia because higher learning institutions and teaching hospitals in Ethiopia have poorly prepared their graduates to conduct scientific trials and clinical research. A recent systematic review reports that there are substantial barriers at the system, organization, and individual level that hamper the conduct of clinical trials in developing countries, and that instituting a system for wider implementation of local investigator-initiated trials would address this problem (37).

Ethical and regulatory system obstacles emerged as the second most important barrier. Prolonged ethical and regulatory review time lead to delays in implementing grants and sometimes led grants to expire before recruitment taking place. They also reported lengthy or ill-defined approval processes, significant bureaucracy, and lack of regulatory staff with expertise in reviewing. Similar findings have been reported in several studies around the globe especially in developing countries reporting (37, 38). Complex and unreasonably strict government regulatory systems could worsen the negative feedback loop between limited research capacity and small numbers of trials conducted. Limited researches in the area were also identified as likely barriers to the conduct of clinical trial for zoonotic diseases in Ethiopia.

Strategies for strengthening the conduct of clinical trials toward zoonotic diseases in Ethiopia need to focus on identifying country-specific problems. The study identified some promoting requirements necessary for successfully conducting a clinical trial for zoonotic diseases that are relevant in the future planning of such a strategy. Most of the promoting strategies reported by participants included the establishment of a well-organized educational system and capacity building. The result of this study reinforces the need for an educational system that integrates portions of clinical trial toward zoonotic diseases to provide students and professionals with a holistic understanding of clinical trial and zoonotic diseases as well as to improve intersectoral collaboration. As most of the Ethiopian scientists are interested to involve in clinical trial studies, there must be some mechanism to address such needs. The higher learning institutions may incorporate modules about clinical trials within their teaching programs. A standard guideline and platform should be established that address conducting clinical trials toward zoonotic diseases, especially on neglected zoonotic diseases by bringing together experts from different disciplines. This finding is supported by a study (39) which stated that capacity building is a basic strategy needed to build good clinical industries. Additionally, policymakers should be involved to support clinical trial studies on zoonotic diseases in this country. Successful promotion of conducting clinical trials toward zoonotic diseases requires government and ministerial office involvement. Raising awareness among decision-makers and policy-makers on the burden of zoonoses in humans and animals may assist in securing political commitment and financial support for conducting clinical trials toward zoonotic diseases. Moreover, participants stressed the importance of sharing experiences from other countries that have a success story in conducting clinical trials toward zoonotic diseases as that could encourage them to get involved in such research. In other countries, there are so many clinical trials conducted on zoonotic diseases, including vaccine trials for anthrax, rabies, and other zoonotic diseases (40–43).

This study has limitations in that it focuses on the perspective of participants included and is not necessarily representative of that of their peers as such these views can not be generalized. In the contrary, its unique approach, with a country-level focus and input from prominent experts around Ethiopia who are influential in their corresponding fields including in the areas of public health, veterinary health, and one health, give the study strength. Further large scale studies involving interviews and surveys of stakeholders including representatives from a range of institutions would be beneficial.

## Conclusion

In Ethiopia, conducting clinical trials on zoonotic diseases deserves due attention. Several challenges hinder conducting clinical trials toward zoonotic diseases. These challenges include ethical and regulatory system barriers, lack of collaboration across different sectors, financial scarcity, lack of human capacity, and administrative related issues. The study illustrates the importance of interdisciplinary collaboration and communication in the field of zoonotic disease control. Strengthening institutional and human resources capacity is a pre-condition to harness effective implementation of clinical trials on zoonotic diseases in the country. In Ethiopia where skilled human resource is scarce, One Health approach has the potential to form multidisciplinary teams to systematically improve clinical trials capacity and outcomes in the country.

## Data Availability Statement

The raw data supporting the conclusions of this article will be made available by the authors, without undue reservation.

## Ethics Statement

The studies involving human participants were reviewed and approved by Scientific and Ethics Review Committee of the Center for Innovative Drug Development and Therapeutic Trials for Africa (CDT-Africa), College of Health Sciences, Addis Ababa University, Addis Ababa, Ethiopia. The patients/participants provided their written informed consent to participate in this study.

## Author Contributions

SB conceived the study, collected the primary data, analyzed, and wrote the first draft. SB, TM, and MG designed the study. TM and MG supervised the data collection and revised the manuscript. All authors approved the final version for publication.

## Conflict of Interest

The authors declare that the research was conducted in the absence of any commercial or financial relationships that could be construed as a potential conflict of interest.

## Abbreviations

AHARI: Armauer Hansen Research Institute
AI: Avian Influenza
CDC: US Centers for Disease Control and Prevention
CDT-Africa: Center for Innovative Drug Development and Therapeutic Trial for Africa
EPHI: Ethiopian Public Health Institute
EWA: Ethiopian Wildlife Authority
FDA: Food and Drug Administration
GCP: Good Clinical Practice
NAHDIC: National Animal Health Diagnostic and Investigation Center
NVI: National Veterinary Institute
RVF: Rift Valley Fever
WHO: World Health Organization.
